# Prospects for γδ T cells and chimeric antigen receptor γδ T cells in cancer immunotherapy

**DOI:** 10.3389/fimmu.2025.1554541

**Published:** 2025-04-30

**Authors:** Lu Wang, Jiaqi Li, Yaping Xuan, Jinrui Zhang, Xiao Wang, Wei Hu, Lei Xiu

**Affiliations:** ^1^ State Key Laboratory of Reproductive Regulation and Breeding of Grassland Livestock, Institutes of Biomedical Sciences, School of Life Sciences, Inner Mongolia University, Hohhot, China; ^2^ State Key Laboratory of Genetic Engineering, School of Life Sciences, Fudan University, Shanghai, China; ^3^ Department of Infectious Diseases, Shanghai Key Laboratory of Infectious Diseases and Biosafety Emergency Response, National Medical Center for Infectious Diseases, Huashan Hospital, Fudan University, Shanghai, China

**Keywords:** γδ T cells, γδ CAR-T cells, cancer therapy, anti-tumor responses, pro-tumor response

## Abstract

γδ T cells, a type of specialized T cell, differ from alpha-beta T cells due to the presence of γ and δ chain surface T cell receptors. These receptors allow them to directly recognize and bind antigenic molecules without the requirement of attachment to MHC or APC antigen presentation. Given their intrinsic properties and functional versatility, γδ T cells are under intensive investigation as carriers for chimeric antigen receptor (CAR) in the context of cancer therapy. In this regard, γδ CAR-T cells have demonstrated great potential to overcome the limitations of antigen recognition with the help of dual antigen identification mechanisms. However, there are still technological challenges that need to be addressed. This discussion focuses on the research status and future development prospects of γδ T cells and γδ CAR-T cells, aiming to provide valuable insights for the follow-up research and practical application of γδ CAR-T cells.

## 1 Introduction

Cancer is now one of the most significant risks to human health globally, with 19.3 million new cases of malignant tumors in 2020. Cancer will become even more prevalent worldwide and its burden will increase, particularly in low- and middle-income nations, as life expectancy rises and the world’s population grows (1).Currently, available cancer treatment strategies include surgery, radiotherapy, chemotherapy, and molecularly targeted drugs. There are also other treatment options available such as biologic therapy and endocrine therapy. Furthermore, in certain situations, adjuvant therapies such as laser therapy, electrochemistry, cryotherapy, microwave thermotherapy, ultrasound thermotherapy, and radiofrequency treatment might also be effective. In recent years, cancer immunotherapy has become another effective cancer treatment due to its rapid development. Tumor immunotherapies are constantly expanding, featuring a diverse range of therapeutic agents and technologies, including tumor vaccines, cellular immunotherapies, T cell-targeting immunomodulatory agents, and immune checkpoint inhibitors (ICIs). Concurrently, ongoing research and development are focused on novel anti-tumor immunotherapies that target a diverse array of mechanisms and targets, such as chimeric antigen receptor T-cell (CAR-T) cellular immunotherapy and bispecific antibodies, all of which are advancing tumor immunotherapy.

In recent years, chimeric antigen receptor T-cell (CAR-T) immunotherapy has demonstrated significant potential in the treatment of acute leukemia and non-Hodgkin’s lymphoma. As research in this area has advanced and technological improvements have been made, the applicability of this therapeutic approach has broadened to include the treatment of autoimmune diseases, solid tumors, cardiac conditions, and human immunodeficiency virus (HIV) infections, among other ailments (2–5). The αβ CAR-T cell-based methodology has transformed the landscape of cancer immunotherapy, but numerous challenges and limitations persist regarding its technical implementation and clinical utilization. Specifically, the preparation of CAR-T cells is costly and entails a lengthy production timeline. Allogeneic αβ CAR-T cells, derived from donor T cells, carry the potential risk of inducing Graft-versus-Host Disease (GvHD), a serious complication resulting from the immune recognition of host tissues by donor cells. In contrast, autologous CAR-T therapy, which uses the patient’s own T cells, does not carry the risk of GvHD. These challenges underscore the pressing need for the development of novel technologies and alternative cell types within CAR-based therapeutic strategies. T cell receptors (TCR) γ and TCR δ dimer-carrying γδ T cells were identified in the 1980s and play a crucial role in the immune system’s fight against infections and tumors. In contrast to αβ T cells, γδ T cells constitute a relatively minor proportion of the circulating T cell population in humans; however, they predominate among the resident T lymphocyte populations found in barrier tissues, including the skin and mucous membranes, and possess a natural homing advantage over αβ T cells, which enables them to respond swiftly to targets, secrete effector cytokines, and effectively infiltrate and operate within the hypoxic conditions characteristic of tumors. Due to their distinctive immunological characteristics, γδ T cells possess the ability to recognize target antigens independently of Major Histocompatibility Complex (MHC) restrictions and can elicit anti-tumor responses without inducing GvHD (6, 7), thereby presenting a highly promising therapeutic avenue.

This paper gives a summary and perspective on the advancement and future possibilities of γδ CAR-T cells.

## 2 Development of CAR-T technology

**Figure 1:**
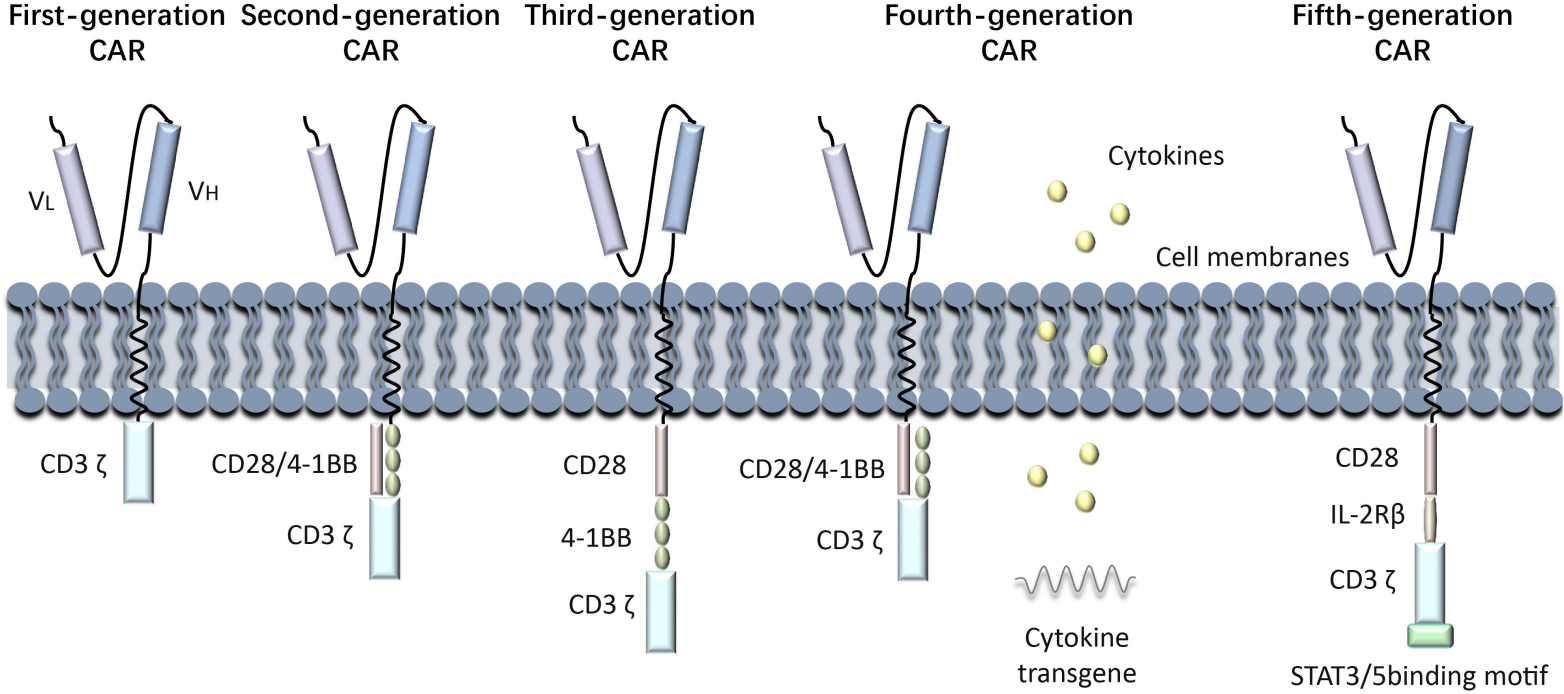
Generations of CAR-T development process. History of CAR-T Immunotherapy: The development of CAR-T cell therapy has progressed through multiple generations, each addressing specific limitations to improve efficacy and safety. First-generation CARs relied solely on CD3ζ signaling, offering limited T cell activation and persistence. Second-generation CARs introduced co-stimulatory domains like CD28 or 4-1BB, significantly enhancing antitumor activity and clinical outcomes. Third-generation CARs incorporated multiple co-stimulatory signals, though their benefits over second-generation designs were modest. Fourth-generation CARs, known as TRUCKs, were engineered to modify the tumor microenvironment by secreting cytokines. Fifth-generation CARs leverage synthetic biology for greater control, precision, and adaptability, incorporating switchable or logic-gated designs.

Chimeric antigen receptor T-cell immunotherapy, also known as CAR-T, is regarded as one of the most promising immunotherapy treatments for cancer. CAR is a synthetic receptor that activates T cells by binding to specific antigens on tumor cells, leading to their direct killing by releasing cytotoxic molecules (e.g. perforin, granzyme B). It also recruits immune cells to kill tumor cells through cytokines release. This treatment can result in long-lasting antitumor effects by developing immunological memory T-cells. Patients can undergo genetic modifications to express CAR on their T cells, which are then infused back into the patient after chemotherapy eliminates endogenous lymphocytes. The process is closely monitored for side effects and effectiveness. The therapy has been available for some time, but it was only recently improved and applied in clinical settings. Israeli immunologist Zelig Eshhar first conceived and validated the concept of CAR-T in 1993 (8), but significant progress was only made in the development of CAR-T in the early 2010s. The first CAR-T therapy, tisagenlecleucel

(Kymriah), was approved by the FDA in August 2017 for the treatment of relapsed or refractory B-cell acute lymphoblastic leukemia (ALL). Since then, advancements in manufacturing processes, such as optimizing T cell activation and transduction efficiency, have improved the scalability and consistency of CAR-T therapies. These improvements have expanded their clinical applications to include other hematologic malignancies, such as diffuse large B-cell lymphoma (DLBCL) and multiple myeloma. Nowadays, research on CAR-T immunotherapy has progressed from the first generation to the fifth generation, showing increased benefits in treating various cancers. over nearly 30 years. The first generation of CAR-T cells involves the fusing tumor antigen-specific single chain antibody fragment (scFv) with the CD3ζ structural domain on T cells, which can be activated by either the CD3ζ chain or tyrosine activation motifs on the FcγR. The CD3ζ chain not only provides activation signals but also signals necessary for lysing tumor cells and controlling the release of IL-2 (9, 10). However, the anti-tumor activity of first-generation CAR-modified T cells was limited *in vivo* due to the lack of co-stimulatory signals, which ultimately led to increased T cell apoptosis. Second-generation CAR-T cells are artificial chimeric T cells that have been engineered to include the CD3ζ structural domain, a CD28 or 4-1BB co-stimulatory molecular fragment, and scFv (11)These modifications enhance the activity and persistence of the cells. Second-generation CAR-T cells exhibit a similar antigen specificity to first-generation CAR-T cells; however, they exhibit markedly enhanced capacity to stimulate T-cell proliferation, produce anti-apoptotic proteins, and secrete cytokines and chemokines in comparison to the first-generation CAR-T cell therapy. CD28 is the more commonly used co-stimulatory molecule, but CD137 (4-1BB) or CD244 can also be used as alternatives for the second-generation CAR-T cells (12, 13). With advancements in technology, scientists have further improved the design of CAR, leading to the development of the third generation of CAR-T cells, that incorporate a classic CD28 and 4-1BB co-stimulating molecule in addition to the features of the second generation. The CAR-T cell contains scFv, two stimulation molecules, and the CD3ζ structural domain, along with co-stimulant like OX40, ICOS, CD27, CD40-MyD88, etc. Previous studies have indicated enhanced and longer-lasting anti-tumor efficacy of these series-built CAR-T cells (14–16). The fourth generation of CAR-T cells, known as “TRUCK” cells, have enhanced the secretion of cytokines, such as IL-12 and IL-2, which play a crucial role in regulating the tumor immune microenvironment. Furthermore, IL-12 attracts innate immune cells like natural killer cells (NK)and macrophages to the tumor’s local immunological milieu, strengthening the anti-tumor actions of CAR-T cells (17). Fifth-generation CAR-T cells, based on second-generation CAR-T cells, have additional co-stimulatory structural domains like IL-2Rβ and STAT3/5 binding motifs, which are able to activate other signaling pathways and provide antigen-dependent cytokine signaling, improving T cell survival, proliferation, and antitumor efficacy in both leukemia and solid tumor models (18) (**Figure 1**).

## 3 γδ T cells and their anti-tumor effects

### 3.1 Ontogeny of gamma delta T cell

T lymphocytes are divided into two groups based on the composition of their TCR dipeptide chains: αβ T cells and γδ T cells. In contrast to αβ T cells, γδ T cells do not exhibit restriction by major histocompatibility complex (MHC) molecules, and the majority of γδ T cells are negative for both CD4 and CD8 markers (19, 20). γδ T cells were first discovered in 1986 by Brenner and others, using the TCR’s γ gene sequence to generate antibodies (21). During the early stages of thymic development, T cell precursors begin to rearrange their β, γ, and δ T cell receptor (TCR) genes. The decision for αβ T cells or γδ T cells lineage is controlled by Notch signaling in combination with TCR signaling intensity. Mice experiments have demonstrated that strong Notch signaling with weak TCR signaling promotes the αβ T cell lineage, while weak Notch signaling with strong TCR signaling leads to γδ T cells development (22, 23). As αβ T cells mature, the proportion of γδ T cells decreases. In the circulating T cells of adult peripheral blood, γδ T cells make up about 4% of all CD3^+^T cells (24). Similarly, in adult mice, the proportion of γδ T cells within the thymus and secondary lymphoid organs is relatively low, comprising approximately 1% to 4% of the total T cell population. Conversely, the proportion of γδ T cells in other mucosal sites is higher, with their proportion in dermal T cells within the skin potentially reaching as high as 50% to 70% (25, 26).

### 3.2 Subtypes of gamma delta T cells

The γδ T-cell antigen receptor TCR is a heterodimer composed of γ and δ chains, encoded by the γ and δ genes. Each chain has variable (V), constant (C), transmembrane, and cytoplasmic regions, encoded by variable (V) and constant (C) regions. The different combinations of V gene fragments provide diversity for the γδ T cells to recognize various antigens. In mice and humans, several Vγ gene fragments have been identified, including Vγ1, Vγ2, Vγ3 and Vγ4 (27, 28). Vγ1 and Vγ2 are expressed in adult T cells, while Vγ3 and Vγ4 T cells are only in the early fetal thymus (29–31).Based on TCR δ chain usage, γδ T cells can be categorized into three subpopulations, including Vδ1 T cells, Vδ2 T cells, and Vδ3 T cells (32). Vδ1 T cells and Vδ2 T cells (referred to as Vγ9Vδ2 T cells) play an important role in the immune response against hematologic tumors. Vδ1 T cells are primarily found in tissues like the dermis and intestinal epithelium, where they act as a defense against epithelial malignancies, and make up a small proportion of the blood (32). Vδ2 T cells can produce perforin, granzyme, interferon γ (IFN-γ) and tumor necrosis factor-α(TNF-α) to directly or indirectly mediate antitumor immunity (33). Vδ3 T cells, the least rarest subset, are primarily located in the liver. Through surface-expressed CD56, CD161, NKG2D, and human leukocyte HLA-DR antigens, Vδ3 T cells participate in the immunological response. In the healthy human body, γδ T cells are mainly distributed in mucous membranes and epithelial tissues, such as skin, digestive tract, reproductive tract and other organs. When activated, they can combat various tumors by releasing cytokines and inducing tumor cell death. γδ T cells are therefore becoming sought-after effector cells for cancer immunotherapy. Furthermore, research has demonstrated that Vδ2 and Vδ3 T cells can assist in B cell differentiation and antibody secretion, as well as promote the maturation of dendritic cells (DCs) into cytokine-secreting antigen-presenting cells (APCs). Vδ2 T cells were found to be more effective than Vδ3 T cells in promoting B cell maturation (34, 35). Apart from the aforementioned three subpopulations, distinct minority subpopulations of γδ T cells have been identified in the peripheral blood of patients diagnosed with lymphoma: Vδ4, Vδ6, Vδ7, and Vδ8 T cells. However, the activation mechanisms and chain pairings of these subpopulations remain unclear (36).

Furthermore, subpopulations of γδ T cells can be classified based on the cytokines they release, with the main subpopulations being IFN γ-secreting T γδ1 cells and IL-17-secreting T γδ17 cells (37). The production of IFN γ by γδ T cells is correlated with intracellular pathogen clearance and antitumor responses, while the generation of IL-17 is associated with host defense against extracellular fungus (38, 39). Recent discoveries have shown that the substances in the tumor microenvironment are responsible for the polarization of γδ cells from γδ1 to γδ17 and γδ Treg. There is a growing interest in γδ Treg, whose function is still unknown, as a potential regulator of cancer or inflammatory diseases (40). Eliminating γδ Tregs has been found to be necessary for effectively treating breast cancer. This is because the IL6-adenosine loop between γδ Tregs and cancer-associated fibroblast (CAF) in human breast cancer accelerates tumor growth (41). Consequently, γδ T cells are now being considered as important effector cells for cancer immunotherapy.

### 3.3 Antigen recognition and activation of γδ T cell

In contrast to traditional αβ T cells, γδ T cells are unable to recognize antigens through conventional peptide-MHC complex recognition mechanisms. However, they possess considerable advantages in the detection and elimination of tumor cells and pathogens, attributable to the extensive diversity inherent in their antigen recognition mechanisms. γδ T cells can recognize a broad spectrum of both protein and non-protein ligands by the TCR.

#### 3.3.1 Phosphoantigen

TCR-dependent γδ T cell recognition of nonpeptide antigens (Ag) has been extensively investigated. The predominant Vδ2 T cells in human peripheral blood, constituting 90-95% of the total number of γδ T cells, are specifically activated by phosphorylated isoprenoid-like precursors referred to as pAg, in response to a broad range of cancers and infectious diseases (42). Previous studies have shown that Vδ2 T cells are activated by isopentenyl diphosphate (IPP) and dimethylallyl diphosphate (DMAPP) (7). Further research identified (E)-1-hydroxy-2-methyl-but-2-enyl 4-diphosphate (HMBPP), a hydroxyl analog of DMAPP, which also activates Vδ2 T cells (43). A recent study explains how γδ T cells detect these pAg within target cells. In the tumor microenvironment, the rapid proliferation of tumor cells stimulates the activity of the mevalonate (MVA) pathway, resulting in the synthesis of elevated levels of cholesterol. This increase in cholesterol concentration subsequently enhances the expression of phosphoantigen (pAg). pAg functions as a “molecular glue,” facilitating the binding between BTN3A1 and BTN2A1, which induces conformational changes in membrane-penetrating proteins, allowing BTN2A1 to bind to the TCR Vγ9 and Vδ2 chains, thereby promoting TCR-mediated activation of γδ T cells (44). Another genome-wide CRISPR screen identified that Adenosine 5’-monophosphate (AMP)-activated protein kinase (AMPK) increases the expression of the BTN2A1-BTN3A complex, leading to enhanced activity of Vδ2 T cells in cancer patients. This regulatory mechanism boosts the T cells’ effectiveness against cancer cells in a metabolic stressful state (45). Abnormalities in the cholesterol metabolic pathway in tumor cells results in the accumulation of isoprenoid pyrophosphate molecules, including DMAPP and IPP. These molecules, along with HMBPP produced by pathogens like Gram-negative bacteria and malaria parasites, activate γδ T cells to release inflammatory cytokines IFN-γ and TNF -α, which kill tumor cells (46).

#### 3.3.2 Cell receptors

γδ T cells can recognize cellular stress proteins and pathogen-associated molecules by expressing NK cell receptors, like NK cell receptor 2D (NKG2D) and DNAM-1 (CD226) (47, 48). They also have natural cytotoxic receptors that bind ligands on hematologic tumor cells, such as NKp30 and NKp44 (49). Additionally, γδ T cells can respond directly to pathogen-associated molecular patterns (PAMPs) through Pattern recognition receptors (PRRs) like Toll-like receptors (TLRs) and nucleotide oligomerization structural domain-like receptors. Some γδ T cell subsets are also capable of recognizing intact proteins or unique PAMPs, including exogenous and endogenous antigenic molecules, through the non-classical MHC pathway (37). Activated γδ T cells exhibit increased expression of TNF-related apoptosis-inducing ligand (TRAIL) and Fas ligand (FasL) (50). FasL interacts with CD95 (also known as Fas or APO-1), activating a caspase cascade that lead to apoptosis in cancer cells (51).TRAIL interacts with death receptors DR4, DR5, decoy receptor 1 (DcR1), and DcR2 (52). Death receptors DR4 and DR5 have a death domain that enables them to initiate cytotoxic signaling when bound to TRAIL (53). Thus, upregulation of CD95 or death receptors DR4 or DR5 in tumor cells can enhance γδ T cell-mediated cytotoxicity. Furthermore, γδ T cells express the Fc receptor (CD16), which can bind to antibodies on tumor cells, allowing them to function as an anti-cancer agent. The anti-tumor immunotherapy potential of γδ T cells is promising due to these features.

### 3.4 γδ T cells in cancer

Several studies have confirmed the link between γδ T cells and the development and outcome of various diseases, including cancers, autoimmune diseases, bacterial and viral infections, and more. The role of γδ T cells in cancer is primarily discussed here.

#### 3.4.1 The direct killing effect of γδ t cells on tumor

There are two main ways in which γδ T lymphocytes can directly kill tumor cells: through trans-antibody dependent cell mediated cytotoxicity (ADCC) and perforin-granzyme, and through FasL and TRAILR-mediated apoptosis (54). γδ T lymphocytes express FasL and tumor necrosis factor-associated apoptosis-inducing ligand (TRAILR), which can induce programmed cell death in tumor cell by binding to Fas-FasL and TRAIL-TRAILR (55). Inhibition of TRAIL can decrease γδ T cell-mediated cytotoxic activity (56). Furthermore, γδ T cells secrete TNF-α and IFN-γ, along with other cytokines. Both IFN-γ and TNF-α play a role in inhibiting cancer growth through various mechanisms, such as direct tumor reduction and prevention of cancer angiogenesis (57, 58). γδ T cells can be induces to release IFN-γ and TNF-α by various stimuli, including TCR agonists, NKG2D ligand, and specific cytokines like IL-12 and IL-18 (59). In the context of tumor immunity, different isoforms of IL-17 produced by γδ T cells have dual effects on cancer, with both pro- and anticancer. Despite its pro-tumorigenic effects and support for angiogenesis, IL-17 can also indirectly suppress tumor progression and metastasis to enhance anti-tumor immune responses (60, 61).

**Figure 2:**
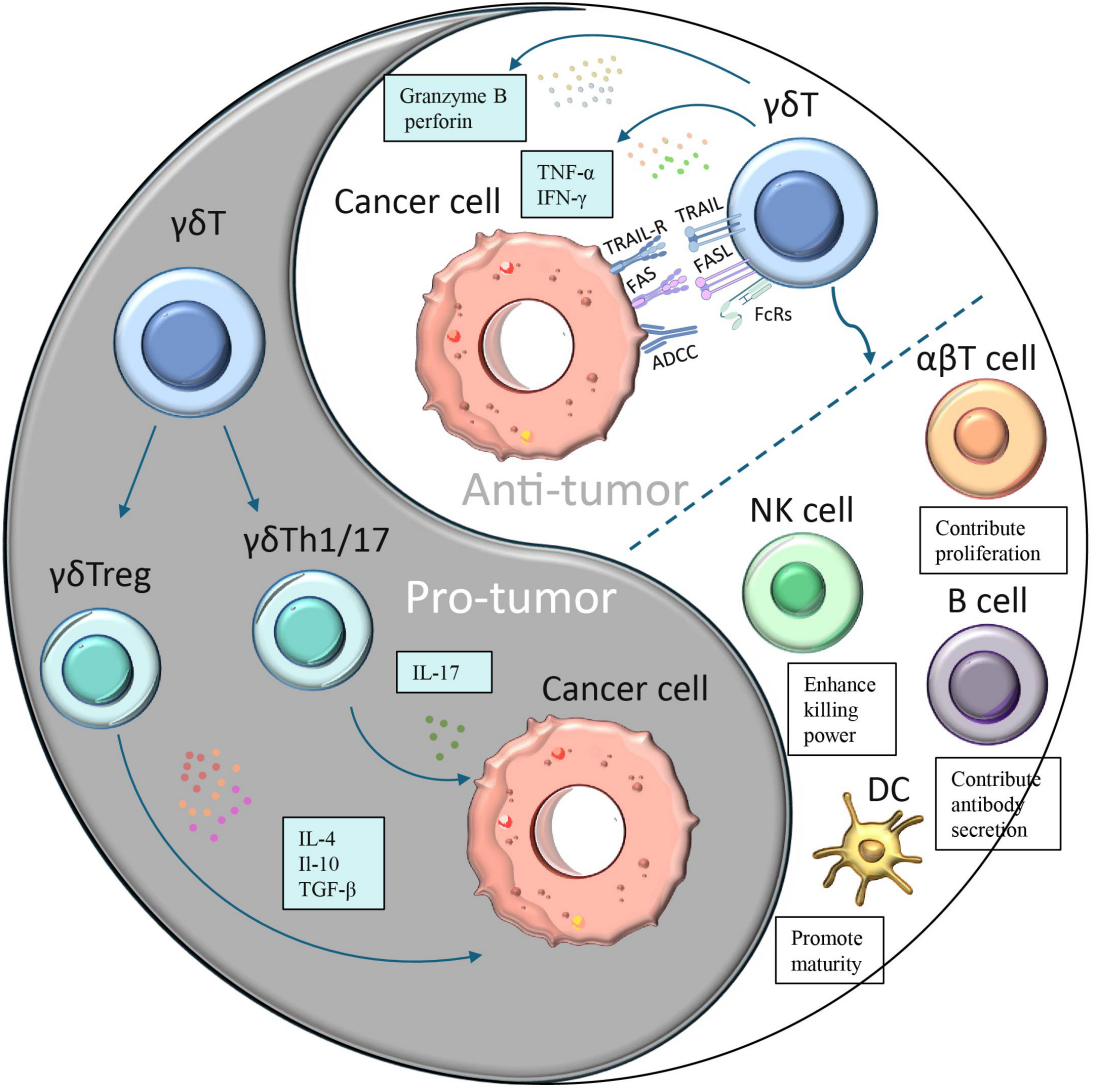
A dual role of γδ T cells in cancer. Different subpopulations of γδ T cells have opposing effects on cancer:(1) γδ T cells polarize to Th1-, Th17- or Treg-like phenotypes in response to specific stimuli and secrete immunosuppressive factors (IL-4, IL-17 or IL-10 and TGF-β), thereby promoting tumor cell proliferation. (2)γδ T cells can produce cytokines such as IFN-γ and TNF-α to directly inhibit tumor growth and block angiogenesis.(3) γδ T cells can directly kill tumor cells through antibody-dependent cell-mediated cytotoxicity (ADCC) and perforin-granzyme pathways.(4) γδ T induces apoptosis of tumor cells through FasL and TRAILR-mediated pathways γδ T cells.(5) γδ T can also generate indirect antitumor effects through their interactions with B cells, DCs, αβ T cells, and NK cells.

Besides their direct cytotoxic effects, γδ T cells can also generate indirect antitumor effects through their interactions with B cells, DCs, αβ T cells, and NK cells (**Figure 2**). Changes in the composition of γδ T cells can affect other immune cells and immunological responses, as these subpopulations of T cells have distinct functions and can interact with each other. Research has indicated that eliminating the Vγ4 γδ T and Vγ6 γδ T subpopulations in nonimmunized mice leads to alterations in peripheral B-cell populations and antibody production (62). Brandes and colleagues found that activated Vδ2 T cells can enhance the proliferation of naïve CD4^+^αβ T cells and accelerate their development into cytotoxic T lymphocytes (CTLs) (63). This is achieved through mechanisms such as the release of TNF-α and IFN-γ, which also stimulate the expression of CD86 and MHC-like molecules on the surface of γδ T cells, promoting DCs maturation (64). Additionally, when CD137L-expressing γδ T cells interact with CD13 co-stimulatory molecules on NK cell surfaces, co-stimulatory signaling can increase NK cell anti-tumor cytotoxicity and enhance their killing ability (65). γδ T cells are capable of processing and presenting antigens, as well as providing co-stimulatory signals that induce immune cells to proliferate and differentiate for effective killing of target cells (66).

#### 3.4.2 Antitumorigenic functions of γδ T cells

Immunotherapy stands as one of the most effective approaches in the fight against cancer today. Despite the challenges posed by the diversity of γδ T cells, these cells have demonstrated tremendous potential in cancer immunotherapy. Research indicates that γδ T cell immunotherapy exhibits positive effects against various types of cancers, including glioblastoma, prostate cancer, colon cancer, lung cancer, liver cancer, kidney cancer, breast cancer, as well as hematologic malignancies such as leukemia, lymphoma, and multiple myeloma, immunotherapy using γδ T cells can extend the span of tumor-bearing mice and has the potential to inhibit tumor growth (67–70).

Among various types of cancer, prostate cancer is the most common malignant tumor in the male reproductive system, with its incidence increasing with age. Research by Mohanad H. Nada et al. revealed that human γδ T cell-based immunotherapy becomes more effective in treating prostate cancer when combined with programmed cell death protein 1 (PD-1) checkpoint inhibitors (71). Specifically, co-culturing activated Vδ2 T cells with the prostate cancer cell line (PC-3) enhances PD-L1 expression in PC-3 cells. More notably, in immunodeficient NOD-*Prkdc* ^scid^ *Il2rg* ^em1^/Smoc (NSG) mice bearing PC-3 tumors, PD-1 monoclonal antibody therapy significantly boosted the immunity of Vδ2 T cells, resulting in almost complete tumor regression after 5 weeks. Colon cancer, a prevalent malignant tumor in the colon, ranks third among gastrointestinal tumors. Wu et al. discovered that Vδ1 T cells isolated from human peripheral blood exhibit high cytotoxicity against colon cancer cells (72). Furthermore, Vδ1 T cells exhibiting exogenous proliferation demonstrated a reduction in tumor growth in transplanted mice with human colon cancer and an extension of survival in mice harboring tumors.

The diversity of γδ T cells extends beyond their role in cancer treatment, also manifesting in associations with other diseases. For instance, research by Alcade demonstrated that women with normal vaginal microbiota have a higher abundance of γδ1 T cells, whereas those with abnormal vaginal microbiota exhibit a greater presence of γδ2 T cells. This diversity in γδ T cells can be utilized to predict the susceptibility of the female vagina to HIV infection (73). Additionally, a recent study based on single-cell sequencing data identified eight hub genes associated with γδ T cells as potential predictive markers for cervical cancer (74).

In the treatment of lung cancer and liver cancer, γδ T cells also play a significant role. The research team at Guangdong-GD Kongming Biotech LLC (GDKM) monitored eight advanced liver cancer patients who had received five or more rounds of cell therapy and ten advanced lung cancer patients who had undergone the same number of treatments. The data revealed that in the liver cancer group, the median survival time for untreated patients was 8.1 months, whereas for those treated with γδ T cell therapy, it reached 23.1 months. In the lung cancer group, the median survival time for untreated patients was 9.1 months, while for those treated with γδ T cell therapy, it was 19.1 months. A comprehensive analysis of the data from both groups indicates that, whether for advanced liver cancer or lung cancer patients, those who received γδ T cell therapy experienced a survival extension of over 10 months. This strongly demonstrates the significant efficacy of γδ T cell therapy in treating advanced liver and lung cancers, its safe applicability in clinical settings, and its remarkable improvement in the immune systems and overall quality of life for advanced cancer patients. This has also become a focal point of attention in the global field of cell therapy (75). Further research has also uncovered more mechanisms of γδ T cells in the treatment of liver cancer. When hepatocellular carcinoma patients have a higher content of γδ T cells in their tumors, their tumors tend to be relatively smaller, and their survival time is prolonged. Although the number of γδ T cells in the blood, liver, and tumor tissues is limited, γδ T cells can transform into γδ tissue-resident memory T (TRM) cells, which help maintain the production of cytokines in liver tumor-infiltrating lymphocytes (TILs) and hepatocytes. This enhances cell survival and antitumor capabilities (76). Furthermore, another study identified a novel protein antigen associated with hepatocellular carcinoma (HCC) called macrophage-stimulating protein (MSP), which can be recognized by γδ T cells. Researchers investigated the function of MSP-activated γδ T cells in HCC, and the results showed that HCC patients exhibited higher serum IFN-γ levels and a higher ratio of peripheral blood γδ T cells compared to the healthy control group. Additionally, the study found that MSP is significantly expressed in HCC and can stimulate γδ T cells to release cytokines and cytotoxic mediators, thereby eliminating HCC cells. Moreover, besides its direct antitumor effects, MSP can also promote the expression of biomarkers associated with antigen-presenting cells (APCs) in γδ T cells, such as MHC-I, MHC-II, CD86, and CD11a. These biomarkers enable γδ T cells to act as antigen-presenting cells capable of stimulating αβ T cells, thereby indirectly exerting an antitumor effect against HCC (77).

Acute myeloid leukemia (AML) is a malignant disease of myeloid hematopoietic stem/progenitor cells. It is mainly characterized by abnormal proliferation of primitive and immature myeloid cells in bone marrow and peripheral blood. The majority of cases have a grave prognosis and are extremely sick. In a recent study by Yue K et al., the prognosis of AML patients following hematopoietic cell transplantation (HCT) therapy and the rate of γδ T-cell recovery were investigated. The study biopsied 103 AML patients and analyzed the recovery of T-cell subsets at different time points after HCT. The findings indicated that early recovery of Vδ2 T cells is a favorable factor for long-term survival in AML patients after haploidentical HCT. The rate of Vδ2 T-cell recovery at day 90 post-HCT was negatively correlated with nonrelapse mortality at years 2 and 5, and the Vδ2 T cell recovery rate at 270 days post-HCT was inversely proportional to the likelihood of AML recurrence at 2 and 5 years. These findings suggest that γδ T-cell-based immunotherapy may help prevent leukemia relapse and infection-related issues in AML patients (78).

#### 3.4.3 Protumorigenic roles of γδ T cells

Numerous experimental data indicate that γδ T cells play a positive role in inhibiting cancer initiation and progression. However, their functions exhibit significant environmental dependency and heterogeneity, which are closely related to their subset differentiation and phenotypic plasticity. Studies have shown that certain γδ T cell subsets, particularly those producing IL-17A, may exhibit pro-tumorigenic functions. γδ T cells can display Th1, Th17, or Treg-like phenotypes depending on the microenvironment. They promote tumor progression by secreting immunosuppressive factors such as IL-4, IL-17, IL-10, and TGF-β, which inhibit the maturation of dendritic cells and the effector functions of γδ T cells, CD4, and CD8 αβ T cells (79). Notably, IL-17 has shown paradoxical roles in various tumor models. On one hand, IL-17 has been demonstrated to activate Vγ6 γδ T cells, promote tumor proliferation, and lead to sustained tissue inflammation and tumor growth (80). In colorectal cancer (CRC) models, IL-17A-deficient mice are protected from tumor invasion (81), and elevated levels of IL-17A are observed in the peripheral blood and tumor tissues of CRC patients (82). Additionally, increased levels of IL-17 are closely associated with poor prognosis and enhanced metastasis in various malignancies, including pancreatic cancer, liver cancer, non-small cell lung cancer, and breast cancer (83). In lung cancer studies, overexpression of IL-17A has been found to accelerate tumor progression by inducing inflammation (84). However, IL-17A was found to play a protective function in the development of lung cancer in a mouse study that used a different hereditary lung tumor model (85).

IL-17 plays a multifaceted and intricate role in tumor progression, promoting tumor development through diverse mechanisms. Firstly, IL-17 directly stimulates tumor cell proliferation by activating the PI3K/AKT signaling pathway, while simultaneously inducing the production of matrix metalloproteinases (MMPs), thereby facilitating tumor invasion and metastasis. Additionally, IL-17 significantly enhances tumor angiogenesis by upregulating the expression of pro-angiogenic factors such as vascular endothelial growth factor (VEGF), IL-6, and IL-8, which provide crucial nutritional support for tumor growth (86–88). Furthermore, IL-17 recruits myeloid-derived suppressor cells (MDSCs) into the tumor microenvironment, fostering an immunosuppressive milieu that attenuates anti-tumor immune responses (89). Notably, MDSCs reciprocally stimulate γδ T cells to produce more IL-17, creating a vicious cycle that perpetuates tumor progression (90). In specific tumor models, the role of γδ T cells becomes even more pronounced. For instance, in the HPV16 tumor protein mouse model, γδ T cells indirectly promote angiogenesis and tumor growth by reducing the expression of epidermal Skint1 and diminishing the population of anti-tumor Vγ5 γδ T cells (91). Moreover, in lung cancer mouse models, the aging process has been found to promote the proliferation of γδ T17 cells. With advancing age, elevated levels of IL-17 in peripheral lymph nodes stimulate the expansion of γδ T17 cells, which subsequently migrate into the tumor microenvironment, exacerbating immunosuppression and tumor progression (92).

The role of γδ T cells in cancer is not unidimensional but exhibits a marked duality. This dual functionality may be attributed to the diverse models and reagents employed in research, as well as the functional heterogeneity of γδ T cell subsets. Such functional heterogeneity and context dependency render γδ T cells both potential therapeutic targets and sources of complexity and challenges in cancer treatment.

## 4 Advancements in the investigation of γδ CAR T cells

### 4.1 Progress in the study of γδ CAR T cells

The characteristics and multifunctionality of γδ T cells have been described above in detail. In light of the numerous advantages offered by γδ T cells, the scientific relevant research on using them as carriers for CAR cell therapy is currently being extensively carried out in the scientific research field. Studies have demonstrated that CAR-modified γδ T cells can secrete IFN-α and upregulate CD69, thereby effectively targeting neuroblastoma and malignant B-cell tumors (93). Further research revealed that CD19-specific γδ CAR T cells, when stimulated by artificial antigen-presenting cells (aAPCs), are capable of proliferation and exhibit significant antitumor activity both *in vitro* and *in vivo* (94). Preclinical data have also confirmed that CAR expression enhances the specific cytotoxic capabilities of γδ T cells against neuroblastoma cells, while these cells can additionally present antigens to αβ T cells, highlighting a dual antitumor mechanism (95). Recent studies have further underscored the enhanced tumor-killing capacity of CAR γδ T cells against both solid and liquid tumors, yielding promising results in murine models (96). However, the limited persistence of CAR γδ T cells *in vivo* restricts their clinical application potential, as a single injection often yields transient effects, necessitating multiple administrations for sustained tumor control. Addressing this challenge, the Du research group successfully overcame these limitations by expanding Vγ9Vδ2 T cells expressing anti-HER2 CAR and anti-CEA CAR, combined with cytokine-induced killer cell populations (97). Combining different CAR cell vectors may enhance immunotherapy effectiveness. Numerous clinical trials investigating γδ CAR T cell therapy are currently underway, including acute myeloid leukemia (NCT03885076), acute T-lymphoblastic leukemia (NCT04702841), B-cell lymphoma (NCT02656147), and solid tumors (NCT04107142). Future research will continue to focus on optimizing the persistence and long-term efficacy of CAR γδ T cells, aiming to achieve greater breakthroughs in tumor therapy.\ Studies of γδ CAR T-based therapies in cancer is summarized in **Table 1**.

**Table 1:** Studies of γδ CAR T-based therapies in cancer.

Tumor type	Effector cells	Mechanism	References
Hepatocellular carcinoma	Glypican-3- CAR-γδ T cells	Exhibit strong cytotoxicity and secrete a variety of chemokines and tissue homing receptors.	(98)
Leukemia	CD19-CAR-γδ T/CD5‐CAR‐γδ T cells	Cytokine secretion, direct target killing and myeloid leukemia cell clearance.	(96, 99)
Renal cell carcinoma	CAR-γδ T cells	Secretion of cytokines such as IFN-gamma.	(100)
Glioblastoma	Allogeneic CAR-γδ T cells	Mediated by γδ T cell receptor and tightly regulated by cellular stress-related NKG2D pathway.	(101)
Prostate cancer	PSCA-CAR-γδ T cells	Pretreatment with zoledronate leads to CAR-independent activation of γδ CAR-T cells, increased cytokine secretion, and enhanced antitumor efficacy.	(102)
Lung cancer	EGFRvIII-CAR-γδ T cells	Recognize and kill EGFRvIII-positive lung cancer cells by releasing cytokines (IFN-γ and TNF-α),and helps to inhibit the growth of transplanted tumors.	(103)
Colorectal cancer	HER2- CAR-γδ T cells	Exhibited strong cytotoxicity and cytokine-secreting ability against CRC cells.	(104)
Breast cancer	FR-α-CAR-γδ T cells	Inhibition of the natural TNBC CDX model by lysing FRα-positive cells and secreting CCL19 and IL-7.	(105)
Oral Cancer	EGFR/MUC1-CAR γδ T cells	Produces local and specific cytotoxic effects on oral cancer cells, while its natural immune-activating properties can recruit other immune cells to further enhance the anti-tumor response.	(106)

### 4.2 Advantages of γδ CAR T-cell therapy

The CAR-γδ T cell therapy utilizes the unique characteristics of γδ T cells to enhance the efficacy and safety of CAR-T cell therapy. Therefore, it combines the advantages of both CAR-T and γδ T cells: 1.γδ T cells do not require major histocompatibility complex (MHC) molecules to recognize antigens. This MHC-independent mechanism enables CAR-γδ T cells to target a broader range of tumor antigens, including those that traditional αβ CAR-T cells may not be able to detect. 2.CAR-γδ T cells possess dual antigen recognition capabilities. They can recognize antigens not only through their engineered CAR but also through their endogenous γδ T cell receptor (TCR). This dual recognition system improves the specificity and efficiency of tumor targeting and reduces the likelihood of off-target effects. 3.γδ T cells serve as a bridge between the innate immune system and the adaptive immune system. They can exhibit a rapid response similar to that of the innate immune system and retain the adaptive memory function. Consequently, CAR-γδ T cells can immediately generate a cytotoxic response upon encountering tumor cells and, at the same time, establish long-term immune memory to prevent tumor recurrence. 4.γδ T cells have the ability to infiltrate solid tumors, whereas traditional CAR-T cells usually have difficulty entering solid tumors. This enhanced tumor homing ability makes CAR-γδ T cells an ideal candidate cell for the treatment of solid tumors. 5.The use of allogeneic (donor-derived) CAR-T cells may lead to GVHD. γδ T cells have been proven to have lower alloreactivity, which makes allogeneic CAR-γδ T cells a safer option for off-the-shelf therapies.

### 4.3 Challenges for γδ CAR T cell therapy

Despite its potential, γδ CAR T-cell therapy still faces various challenges. One of the main challenges facing γδ CAR T-cell therapy is the difficulty in obtaining enough γδ T-cells for therapy, both *in vivo* and *in vitro (* 107, 108). Another issue is the tendency for γδ T cells to convert to a depleted phenotype, resembling αβT cells, after prolonged exposure to antigens, reducing their ability to fight cancer. This conversion leads to a decreased in immune checkpoint proteins, cytokine production, and effector functions (109). While γδ T cell hold promise for cancer therapy, their limited durability *in vivo* allows tumors to grow and necessitates multiple infusions of γδ CAR T cells to enhance their effectiveness (110).

Currently, researchers are also exploring these problems above, about γδ T cell expansion although there is no mature method to address these issues, one study demonstrated that Vγ9Vδ2 T cells from glioblastoma (GBM) patients could be expanded using zoledronic acid and interleukin-2 (IL-2). Following this expansion, the cells underwent gene transduction with methyl guanine DNA methyltransferase (MGMT), which was subsequently used in conjunction with temozolomide (TMZ) chemotherapy, and delivered directly to the area surrounding the residual tumor via a Rickham catheter. This approach offers improved opportunities for the preparation and maintenance of Vγ9Vδ2 T cells and allows continuous local administration of cells (111). And recently IN8bio has unveiled its advanced γδ T cell engager (TCE) platform, which is aimed at the treatment of oncology and autoimmune diseases. This innovative γδ TCE specifically targets and enhances the proliferation of Vδ1^+^ and Vδ2^+^ T cells. It is developed to overcome a significant limitation associated with existing γδ TCEs, namely the inadequate quantity of effector cells necessary to achieve a real clinical effect. For CAR-γδ T products, another aspect that needs to be focused on is their durability *in vivo*. The third-generation CAR-T cells developed in recent years are designed to introduce two or more co-stimulatory molecules to realize the coordinated promotion of the function of CAR-T cells. Thus, one of the solutions to the durability problem is to optimize the structure of CAR, such as adjusting the signaling domains and co-stimulatory domains of CAR, to enhance its proliferation ability and durability *in vivo*. In addition to CAR structure optimization, gene editing strategies - knocking out certain negative regulators or overexpressing certain survival-related genes can also be used to reduce the clearance of γδ T cells by host cells *in vivo*, thereby enhancing their persistence. In addition to this there are clinical trials aimed at designing and developing strategies to improve the persistence of CAR-T cells *in vivo* independently of CAR-T cells. For example, regular infusion of T-cell antigen-presenting cells (T-APC) and regular activation of anti-CD19 CAR-T cells after patients have remission were used to determine whether intermittent stimulation could reactivate and numerically expand CAR-T cells and prevent antigen-positive relapses (NCT03186118). It is important to note that the viability of CAR-γδ T cell products also exhibit a significant correlation with their therapeutic effectiveness. A recent study has remarkably enhanced the *in vivo* activity and proliferative capacity of γδ T cells through the utilization of garlic-derived nanoparticles, offering substantial insights for related research (112). The application of nanotechnology or drug delivery systems to augment the functionality and persistence of CAR-γδ T cells may emerge as a novel and potent strategy in the combat against cancer.

## 5 Summary

The field of immunotherapy is currently undergoing a new phase, with CAR-T cell therapy emerging as a promising approach for the treatment of malignant tumors. γδ T cells are a group of “atypical” T lymphocytes that can recognize a variety of tumor antigens in a non-MHC-dependent manner, allowing them to be activated to exert their tumor-killing functions. In addition, γδ T can also enhance the response of other immune cells by producing cytokines or chemokines; or as antigen-presenting cells, inducing activation of T lymphocytes. These inherent characteristics and versatility of γδ T cells enable them to become excellent candidates for CAR cell therapy. Furthermore, γδ CAR T cells have fewer toxic side effects and do not cause GVHD, which has broad prospects in tumor immunotherapies. However, CAR-T cells also face several challenges, such as limited persistence, low circulating levels, difficulties in preparation and transfection, etc. Researchers are committed to improving the efficacy of CAR-T cell therapy. They are constantly enhancing the quality of CAR - T cell products, refining the structural design of CARs, and establishing a sound quality control system covering all aspects of CAR - T cells, from pre-treatment, and infusion to *in-vivo* monitoring. Moreover, they are exploring the *in-vivo* biological characteristics of CAR-T cells and the mechanisms of tumor immune evasion, developing new targeted drugs and combination treatment regimens to further improve the clinical efficacy of CAR-T cell therapy and reduce its side effects. There is every reason to believe that with the continuous progress of molecular biology and immunology, cell immunotherapy led by CAR-T cells is bound to change the existing treatment paradigm.
